# Alzheimer's Gone Viral: Could Herpes Simplex Virus Type-1 Be Stealing Your Memories?

**DOI:** 10.7759/cureus.11726

**Published:** 2020-11-27

**Authors:** Rhutuja Khokale, Ayesha Kang, Keri-Ann R Buchanan-Peart, Maxine L Nelson, Oluwatayo J Awolumate, Ivan Cancarevic

**Affiliations:** 1 Neurology, California Institute of Behavioral Neurosciences & Psychology, Fairfield, USA; 2 Internal Medicine, California Institute of Behavioral Neurosciences & Psychology, Fairfield, USA

**Keywords:** herpes simplex and alzheimer's disease, antiviral agents and alzheimer's disease, alzheimer's disease/virology, alzheimer's disease and herpes simplex virus type 1, alzheimer's disease, alzheimer's disease and hsv1

## Abstract

Alzheimer's disease (AD) is one of the principal causes of disability and morbidity. It is one of the most expensive illnesses. Despite this, there are no significant data regarding its etiology and optimal treatment. This review concentrates on the viral hypothesis of AD. After a comprehensive PubMed literature search, we analyzed the studies associating herpes simplex virus type-1 (HSV1) infection to AD from the previous 10 years. Molecular mechanisms whereby HSV1 induces AD-related pathophysiology, including neuronal production and accumulation of amyloid-beta (amyloid-β), abnormal phosphorylation of tau proteins, impaired calcium homeostasis, and autophagy, are addressed. The virus also imitates the disease in other ways, showing increased neuroinflammation, oxidative stress, synaptic dysfunction, and neuronal apoptosis. Serological studies correlate HSV1 infection with AD and cognitive impairment. A causal link between HSV1 and AD raises the concept of a simple, efficient, and preventive treatment alternative. Anti-viral agents impede brain degeneration by preventing HSV1 spread and its replication, decreasing hyperphosphorylated tau and amyloid-β; thus providing an efficacious treatment for AD. We also mention brown algae, intravenous immunoglobulin (IVIG), and a synthetic drug, BAY57-1293, with anti-viral properties, as options for treating AD. We want to recommend future researchers to look for more affordable, non-invasive, and swifter techniques to identify HSV1 in the brain and assist in the early detection and prevention of AD.

## Introduction and background

"Alzheimer's disease locks all the doors and exits. There is no reprieve, no escape." 

-Patricia Reagen Davis.

The dominant subtype of senile dementia in the world is Alzheimer's disease (AD) [1-6]. In 2015, it accounted for 18-20 million cases globally [7-8]. In 2014, the United States (US) accounted for approximately five million cases, and epidemiologists expect a rise to nearly 14 million by 2060 [9]. Among the elderly, it is the sixth leading cause of death in the US [9]. The median annual total expenditure for one patient having advanced AD is estimated to be more than $50,000 [10]. By 2040, the cost of treating AD is expected to rise from around $215 billion to more than $500 billion annually [9]. The economic burden of AD in the US rounds up to 1.09% of global GDP [3]. Data have shown an expenditure of $604 billion worldwide in 2010 [8,11].

AD is a progressive neurodegenerative disorder of multifactorial etiology, causing irreversible pathological changes in the brain [4,7,11-16]. Macroscopically, this appears as atrophy of the brain starting from the locus coeruleus, entorhinal cortex, and hippocampus of medial temporal lobes and gradually affecting the inferolateral temporal cortex and nucleus basalis of Meynert [2,10-11]. Microscopically, the changes are seen as amyloid plaques (extracellular), neurofibrillary tangles (NFTs), and neuropil threads (intracellular) [2-3,5-8,10-11,14-15,17-19]. These are the characteristic pathological hallmarks of AD [2-3,5-8,11,15,17-19]. Neuritic/senile amyloid plaques are composed of amyloid-β protein obtained due to the proteolysis of amyloid-β precursor protein (APP) [2-3,7,10,16,19]. APP is a transmembrane protein involved in neurite growth, synaptogenesis, and transmembrane signal transduction [11,19]. NFTs are made up of abnormally hyperphosphorylated tau protein and reside inside the nerve cell bodies, whereas neuropil threads lie within dendritic processes [2-3,7-8,11,14]. Tau is a neuronal stabilizing microtubule-associated protein and becomes pathological solely in its hyperphosphorylated form [7-8,10,19]. Other pathologies seen in AD include neuroinflammation, degeneration of neurons, and loss of synapses [2,14]. Most of these pathological changes appear well before any symptoms are noticed [18,20]. In approximately 80% of the patients, the cognitive changes follow a predictable path, starting with memory impairment to verbal and visuospatial deficits, ultimately leading to executive dysfunction [2,10,15]. Additional symptoms include behavioral abnormalities, intellectual impairment, and progressively deteriorating cognitive function leading to dementia [2,4,11,14-15]. Symptoms gradually worsen, and ultimately, the ability to communicate and perform daily living activities is severely impaired, resulting in a loss of independence [2,10,15]. The initiation and progression of AD appear to be affected by genetic predisposition and a myriad of environmental and lifestyle factors [7].

Early-onset AD (EOAD) and late-onset AD (LOAD) are two classified forms of AD [2,15]. EOAD presents in patients below 60-65 years and comprises approximately 1%-6% of all AD cases [2,15]. Autosomal dominant genetic mutations in either APP, PSEN1 (presenilin1), or PSEN2 (presenilin2) genes links to EOAD, all of which affect the breakdown of APP and leads to the formation of amyloid-β plaques, a trait of AD [2,10,15,18]. LOAD accounts for the majority of AD cases (approximately 95%) and usually occurs after 60-65 years [2,15]. The exact etiology of LOAD is unknown, but certain risk factors have been identified [2,15,19]. The genetic risk factor of utmost importance for AD is the apolipoprotein-E epsilon4 allele (APOE-ε4) [2,4,5-8,11,15,17,19-26]. One allele doubles or triples the risk, whereas two alleles increase the risk by 16 manifolds [10]. Several other factors predisposing to the development of AD include traumatic brain injury, diabetes mellitus, hypertension, dyslipidemia, vascular infections, obesity, physical inactivity, and smoking [2,10,15,21]. Lower socioeconomic status, poverty, minority status (African-American and Hispanic ethnicity in the US), and lower educational attainment are other potential risk factors [2]. Presently, there is no reliable cure for AD [5,7-8,10,17,27]. Treatment options for AD include cholinesterase inhibitors and memantine [10,17]. Cholinesterase inhibitors like donepezil, galantamine, and rivastigmine act to increase acetylcholine availability at synapses for better communicability amongst neurons [10,28]. These drugs have limited efficacy and do not alter the course of the disease [17]. Many recent studies/trials have failed to provide an effective cure for AD, including anti-amyloid treatments like solanezumab [17-18]. Current treatments for AD are merely palliative, and thus there is an urgent need for medications that delay disease progression [27].

Herpes simplex virus type-1 (HSV1) is a highly neurotropic, double-stranded deoxyribonucleic acid (DNA) virus [2-4,7,11,14-17,19,21,25,29-31]. It is a ubiquitous pathogen that affects 80%-90% of the US population by the sixth decade, with persisting serum antibodies [2-3,7,11,14,17,24]. It primarily infects the oral, corneal, and dermal epithelium causing vesicular lesions [4,11,14-15]. It can escape the immune system and become dormant in the sensory ganglion [4,7,15,19,21,24,30]. The trigeminal ganglion is the primary reservoir for HSV1 during dormancy [2,7,11,15-17,20,24]. More than 90% of US adults have HSV1 DNA in their trigeminal ganglion [20]. It can establish a lifelong infection and spread readily among humans [2,15,17]. Prolonged latent periods and episodic recrudescence characterize HSV1 infection [2,15-16]. Upon reactivation, the virus can travel through the sensory ganglia back to the epithelium and cause herpes labialis, commonly known as cold sores [2,4,7-8,11,15-17,22]. In approximately 25% of individuals infected with HSV1, the virus reactivates and migrates to the brain, causing herpes simplex encephalitis (HSE) [4,11,17,21,29-30]. HSE is the most prevailing form of sporadic viral encephalitis [2,14,21,32-33]. Surprisingly, the APOE-ε4 genotype is a risk factor for HSV1 infection (cold sores) [5,8,11,14,17,24,27,32-33].

This review examines evidence suggesting HSV1 as a causative agent of AD. We studied the literature focusing on viral characteristics of HSV1, the mechanisms this virus uses to enter the brain and cause neuropathological changes, potential treatment alternatives, and the genetic background of AD. We used PubMed to identify relevant papers using the keywords: ("Herpes Simplex"[Mesh]) AND "Alzheimer Disease"[Mesh], ("Antiviral Agents"[Mesh]) AND "Alzheimer Disease"[Mesh], ("Alzheimer Disease/virology"[Mesh], Alzheimer's disease and herpes simplex virus type-1. We included the studies published within the last 10 years.

## Review

The entry of herpes simplex virus type-1 into the brain

Aging is the principal risk factor for AD [11]. Cell-mediated immune response by CD8+ T lymphocytes and interferon-gamma inhibits viral reactivation from latency [15]. Immunosenescence affects this response, making the brain vulnerable to infectious agents [14-15]. HSV1 has been proposed as a potential risk factor in the development of AD [1-4,8,11,13-14,17-19,21,24-25,29,31,33-39]. A meta-analysis by Steel et al. concluded that there is an increased risk for AD when HSV1 is present in the brain compared to controls (OR 1.38; 95% CI 1.14-1.66) [35]. The numerous factors mentioned in Table 1 trigger viral reactivation from latency.

**Table 1:** Risk factors responsible for activation of latent HSV1 HSV1 – Herpes simplex virus type-1

Sr. No.	Causes of viral reactivation	Author (Reference)	Year of Publication
1.	Generalized/peripheral infection or inflammation.	Harris et al.[7]	2015
		Itzhaki et al. [8,22,32,33]	2012,2014,2017,2018
		Wozniak et al. [27]	2011
2.	Morbid state and immunosenescence.	Harris et al. [15]	2018
		Mangold et al. [19]	2019
		Itzhaki et al. [32]	2017
		Eimer et al. [37]	2018
3.	Impaired integrity of the blood-brain barrier (BBB).	Devanand et al. [17]	2018
		Mangold et al. [19]	2019
		Eimer et al. [37]	2018
4.	Stress and immunosuppression.	Olsson et al. [5]	2016
		Harris et al. [7,15]	2015,2018
		Piacentini et al. [11]	2014
		Devanand et al. [17]	2018
		Ball et al. [20]	2012
		Itzhaki et al. [22,32,33]	2014,2017,2018
		Tudorache et al. [23]	2017
		Lathe et al. [24]	2019
		Wozniak et al. [27]	2011
		Epstein et al. [36]	2020
		Rizzo et al. [40]	2020
5.	Head trauma.	Wozniak et al. [27]	2011

Upon reactivation, the virus can follow an anterograde or retrograde path [2,17]. In retrograde fashion, there is axonal transport of HSV1 particles, which infiltrate the locus coeruleus progressing to the temporal lobe, particularly the hippocampus and entorhinal cortex [7,17,20]. Another mechanism is the intraneuronal flow of viral particles along the trigeminal nerve branches that supply basal meninges or the olfactory pathway [14,20]. Martin et al. also provided evidence of various envelope glycoproteins like gD, gB, and gE in the transsynaptic spread of viruses [14]. Thus, the virus can access synaptically linked neural circuits [14]. The dendritic nerve terminals of olfactory receptor neurons are exposed directly and, therefore, the macromolecules enter freely and transport across the synapses [17]. These olfactory receptors further synapse onto the olfactory bulb's mitral cell neurons, which project onto the entorhinal cortex, amygdala, and hippocampus [15]. Animal studies by Harris et al. demonstrated the use of this pathway by HSV1 [15]. They also identified viral DNA in olfactory bulb samples of the human brain using polymerase chain reaction (PCR) [15]. They found impaired olfactory function associated with increased incidents of mild cognitive impairment (MCI) and AD [15]. The study also found neurodegenerative pathology in the olfactory bulb and tract in early AD [15]. Lastly, the virus spreads via the bloodstream due to the disruption of the blood-brain barrier (BBB) [3-4,11,17,41]. The upregulation of neuroinflammatory markers and early neurodegeneration accompanies viral reactivation [7]. Thus, recurrent HSV1 reactivation in the brain could lead to AD-associated neurodegenerative processes [32].

These studies demonstrate a relationship between HSV1 and AD. A physiological decline in the immune system is a common reason for infection by HSV1, which enters the brain to reactivate later and cause further damage. This literature can be explored further to identify the population at risk for HSV1-induced AD and encourage researchers to find a preventive measure.

Pathological changes in the brain by herpes simplex virus type-1

Neuroinflammation and Oxidative Stress

**Figure 1:**
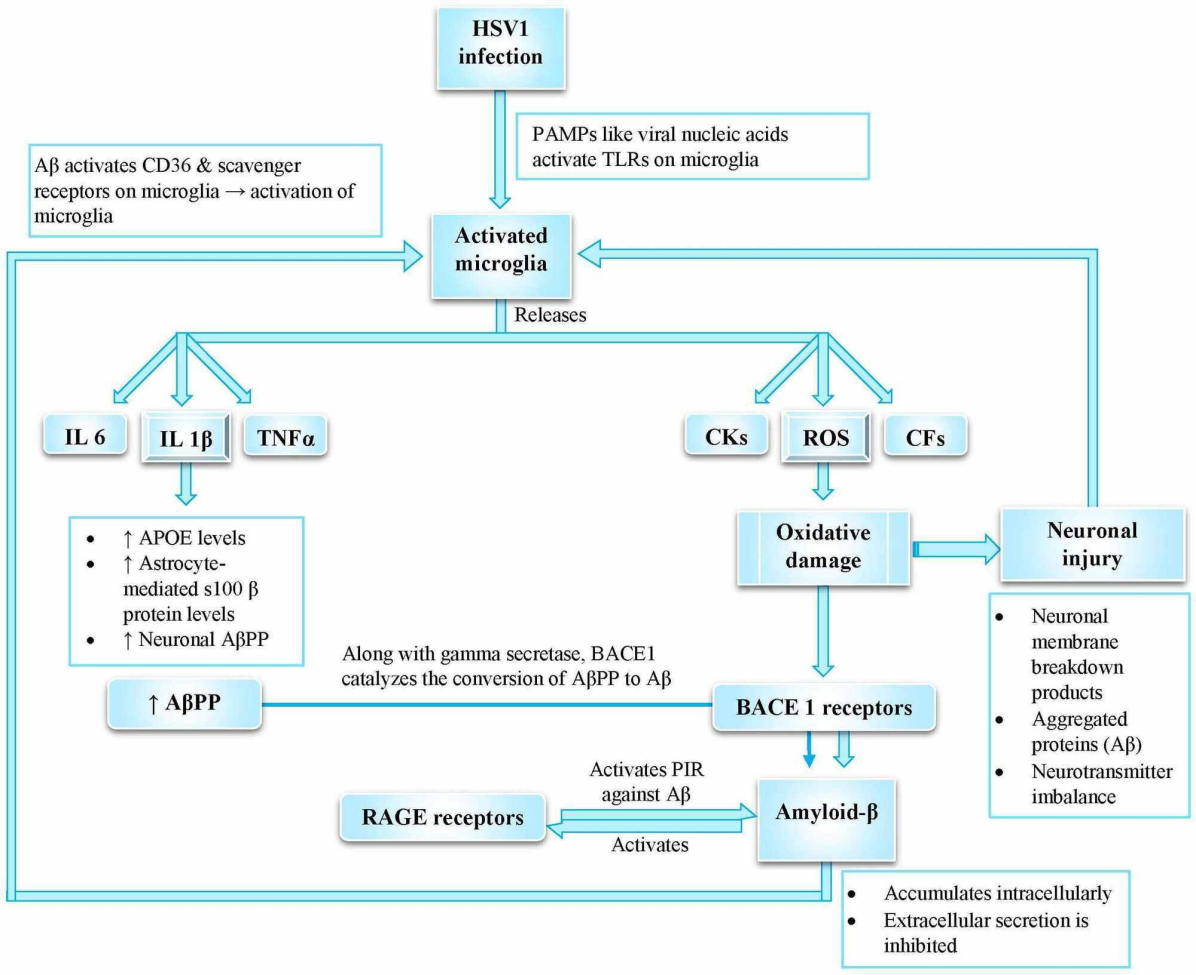
Viral nucleic acids on HSV1 interact with TLRs on microglia, leading to microglial production of pro-inflammatory cytokines Studies support ROS as a mediator of neuroinflammation and AD pathogenesis. Interactions between HSV1 and oxidative stress promote neurodegenerative processes found in early AD. Key: HSV1 – Herpes simplex virus type-1, TLRs – Toll-like receptors, PAMPs – Pathogen-associated molecular patterns, IL 6 – Interleukin6, TNFα – Tumor necrosis factor α, IL1β – Interleukin 1β, CKs – Cytokines, CFs – Complement Factors, ROS – Reactive oxygen species, AβPP – Amyloid β precursor protein, BACE1 – Beta-site amyloid precursor protein cleaving enzyme1, RAGE – Receptor for advanced glycation end-products) References: [2,7,14-15,18,20,32,42]

Microglia function as innate immune cells (resident macrophages), providing defense against pathogen invasion [15]. Upon HSV1 infection (Figure 1), the activated microglia release pro-inflammatory molecules, leading to increased formation of reactive oxygen species (ROS) and reactive nitrogen species (RNS) [7,15]. These reactive species cause oxidative damage, further promoting neurodegenerative processes [7]. An uncontrolled inflammation and amplified cytokine cycle induce neuronal injury, apoptosis, and chronic disease progression [7,15].

Neuronal injury, neuronal membrane breakdown products, cytosolic compounds, and glutamate excess further activate microglia [7]. Thus, overworked microglia damage the neurons [18,32]. This vicious cycle repeats itself to establish an inflammatory milieu [7,15]. HSV1, along with oxidative stress, potentiates the accumulation of intracellular amyloid-β and inhibits its secretion to the extracellular medium [7,42]. Harris et al. concluded that interactions between cytokines and the brain after crossing the BBB is the likely mechanism of neuropathology and brain dysfunction [15].

**Figure 2:**
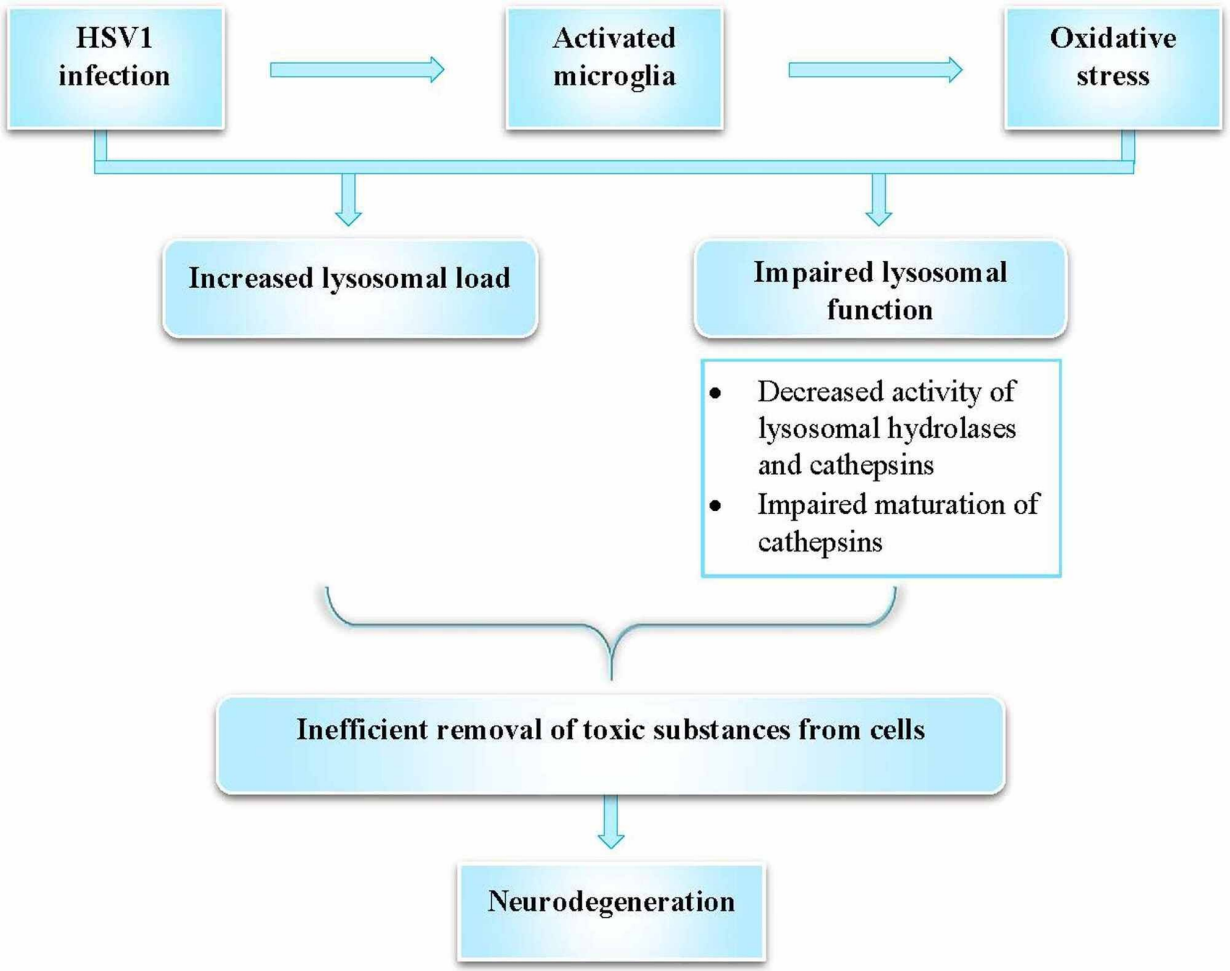
Neurons are particularly susceptible to lysosomal damage Impaired lysosomal function due to HSV1 and oxidative stress leads to the accumulation of lysosomes and decreased functionality of lysosomal proteins HSV1 – Herpes simplex virus type-1 Reference: [33]

Another aspect highlighted by Itzhaki et al. linking HSV1 to AD, is lysosomal impairment due to interactions between HSV1 infection and oxidative stress leading to the accumulation of toxic substances, further accelerating neurodegenerative changes (Figure 2) [33].

The above changes are known to occur early in AD development, thus supporting the role of HSV1 in AD [33].

APOE and phosphatidylinositol binding clathrin assembly protein (PICALM) are essential susceptibility genes in AD [7,33,43]. These susceptibility genes are linked with the HSV life cycle and correlate to cellular entry, intracellular transport, and APP processing [7,43]. Some of these susceptibility genes can lead to abnormalities in autophagy [33]. HSV1 inhibits the homeostatic process involved in the turnover/elimination of cytoplasmic components, damaged organelles, and protein aggregates, thereby modulating the host autophagy [4,15]. This mechanism also contributes to the deposition of amyloid plaques within the brain [4,15,42]. Mawanda et al. state that severe, recurrent, or chronic systemic infections can permanently damage the central nervous system (CNS), ultimately manifesting as cognitive impairment or dementia [2].

Neurofibrillary Tangles (NFTs)

HSV1 induces glycogen synthase kinase 3-beta (GSK3-beta) and protein kinase-A (PK-A), enzymes that cause tau phosphorylation at several sites [2,15,17,19,27,36]. A study by Harris et al. on neuroblastoma cells and murine neuronal cultures infected by HSV1 demonstrates the same [7]. In vitro and animal studies by La Rosa et al. correlate with the above findings [30]. Research on mouse neuronal cultures infected with HSV1 by Mawanda et al. displayed abnormal microtubule dynamics, tau hyperphosphorylation (P-tau), and significant neurite damage, ultimately resulting in apoptosis [2]. Harris et al. and Santana et al. suggest that HSV1 induces apoptosis using infected cell protein 34.5 (ICP 34.5), which dephosphorylates eukaryotic initiation factor 2α (eIF2α) to block both the shutdown of host cell protein synthesis and apoptosis [15,42].

P-tau occurs in the activation pathway of the apoptotic process as a requirement for all changes at the cellular level that ends with the generation of apoptotic bodies [14]. They suggest that apoptotic processes and the neurodegeneration of the cytoskeleton are closely associated and occur due to various neurotoxic stimuli [14]. They found that HSV1 induces the hyperphosphorylation of Alzheimer-type tau epitopes, presenting a close analogy to the hyperphosphorylation processes described in neurodegenerative diseases [14]. Harris et al. and De Chiara et al. list the events after P-tau as conformational alterations forming paired helical filaments (PHFs) or NFTs, associated microtubule destabilization, synaptic damage, and neurodegeneration [15,29]. Viral kinases contribute to the occurrence of P-tau due to cross-species kinase promiscuity, whereby both human and viral kinases phosphorylate both human and viral proteins [17,20]. Another reason is the amino acid homology between human tau and HSV virus protein-22, the target of kinase UL13, which phosphorylates human tau [17,20].

Amyloid Plaque

The migration of new viral particles inside an infected cell requires interactions among HSV1 capsid proteins and APP [4,8,17,22]. Interaction between amyloid-β and HSV1 protein gB leads to HSV1 infectivity impairment by preventing the virus from fusing with the plasma membrane [34,44]. In early HSV1 infection, amyloid-β production plays a protective role in limiting it [3,19,29,37,40,44]. Following repeated viral reactivations, amyloid-β production switches from being defensive to becoming neurotoxic [19,29,32]. Eimer et al. list the possible factors mediating this switch as pathogen virulence and persistence, host genetics, and environmental factors [37]. Aβ peptide is overproduced to protect against latent HSV1 infection, leading to AD progression by contributing to amyloid plaque formation [2-3,34]. Overproduced Aβ leads to synaptic dysfunction, causing cognitive impairment [38]. In HSV1 infected neurons, there is an Aβ-dependent reduction in the expression of the presynaptic proteins associated with the diminished synaptic transmission; Piacentini et al. have first documented this [38]. In a study by De Chiara et al. on cultured mouse cortical neurons, HSV1 induced Aβ accumulation impaired synaptic function proving to be neurotoxic [29].

**Figure 3:**
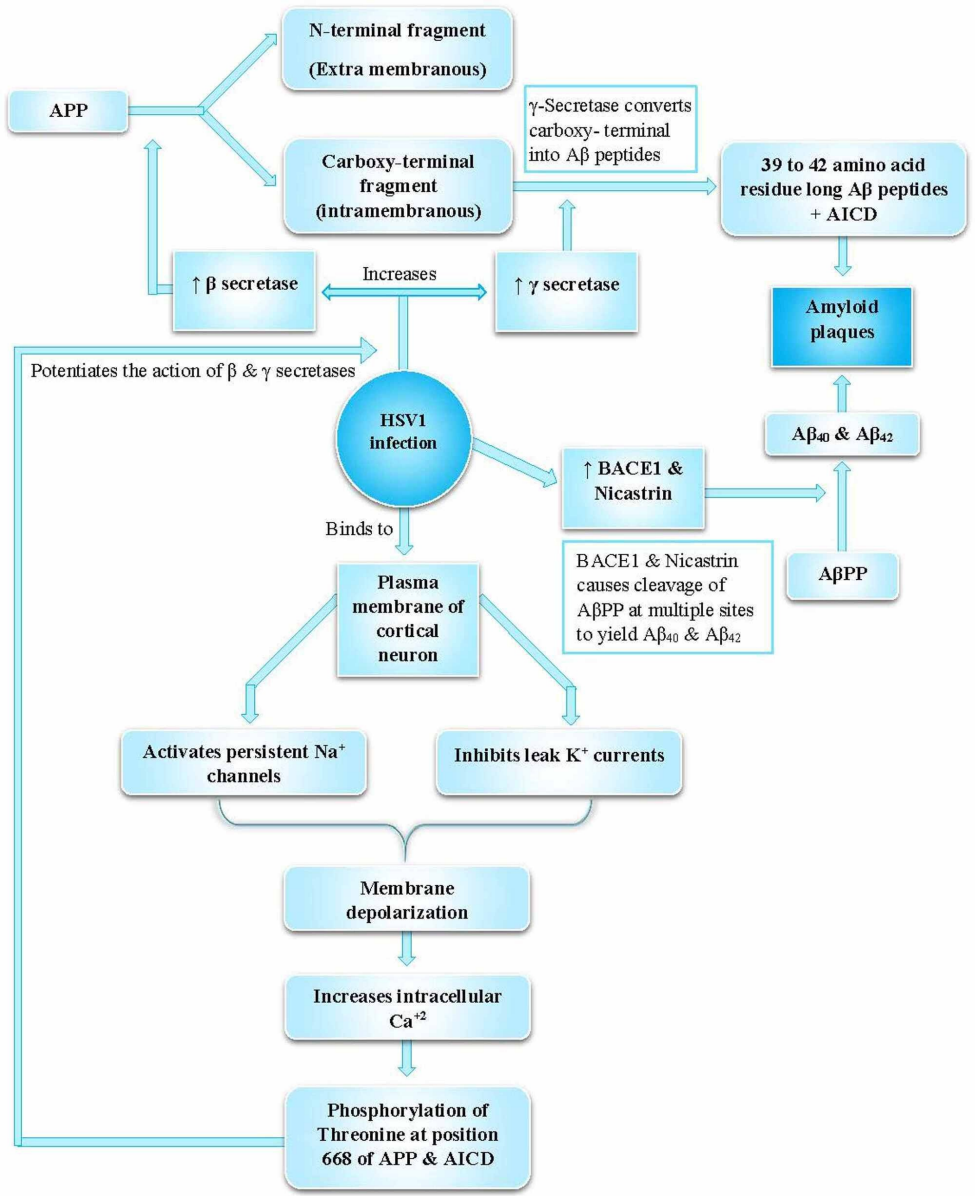
HSV1 infection increases β and γ secretase, which participate in the amyoidogenic pathway to ultimately form Aβ peptides and AICD In the amyoidogenic pathway, α and β secretases break down APP into N- and carboxy-terminal fragments. Aβ40 and Aβ42 primarily form the classical amyloid plaques seen in AD. The non-amyoidogenic pathway is made up of α and γ secretase. Key: HSV1 – Herpes simplex virus type-1, APP and AβPP – Amyloid-beta precursor protein, AICD – APP intracellular domain, BACE1 – Beta site APP cleaving enzyme References: [2,7,11,13,15-18,29,36,38,41]

Studies on mice infected with HSV1 demonstrated that HSV1 reactivations triggered amyloid-β aggregation [29]. This correlated with cognitive impairment in them [29]. A parallel investigation validated that the accumulation of AD hallmarks in the same mice's brain displayed impaired memory in behavioral tests [29]. These findings demonstrate that HSV1 infection of neuronal cells can generate multiple APP fragments with neurotoxic potentials (Figure 3) [29].

Makin et al. showed that the aggregation of amyloid-β triggers a cascade of disease-causing events such as inflammation, NFT formation, synapse dysfunction, and cell death, leading to dementia [18]. Carter et al. state that AD susceptibility genes like APOE, apolipoprotein A1 (APOA1), clusterin, alpha 2-macroglobulin, insulysin, and caspase-3 adhere to HSV1 and viral binding complement components, C3 and complement receptor-1 (CR1), which are involved in the clearance/degradation of amyloid-β [43]. 

Harris et al. imply that amyloid plaque results from immunologic warfare between host and HSV1 [15].

Summary of Pathological Changes

The reactivated virus causes confined local damage via inflammatory and oxidative effects [15,32]. An increase in the intracellular levels of amyloid-β and a decrease in APP and P-tau follow [4,17]. This further accelerates the deposition of amyloid plaques and NFTs, which are the main components of AD [13,17]. HSV1 infection induces early upstream events that eventually lead to Aβ deposition and P-tau and thus superimpose our speculation that HSV1 is a possible risk factor for AD [39]. Pathogen-induced inflammation and CNS accumulation of amyloid-β damage the BBB, contributing to the pathophysiology of AD [7]. Thus, a vicious cycle of uncontrolled neural inflammation and neurodegeneration ensues [7,11]. Thus, data show that reactivation of HSV1 infection causes AD [12-13,17,20-21,25,29,42,45].

Repeated cycles of HSV1 reactivation triggers chronic inflammation causing synaptic loss, leading to cognitive deficits. HSV1 infection induces upregulation in the expression of principal amyloid-β processing components leading to its deposition in the brain. Over-production of amyloid-β to contain HSV1, combined with decreased clearance of its aggregates due to aging, is neurotoxic and results in insoluble plaques. HSV1 infection increases the expression of enzymes involved in tau phosphorylation: GSK3β and PKA, resulting in tau hyperphosphorylation in infected neurons. This leads to neuronal cytoarchitectural changes, which affect synaptic stability and cognitive function. The above data demonstrate that it is, in fact, the reactivation of the virus that is responsible for the initiation of early AD changes.

Epidemiological, genetic, and serological link between herpes simplex virus type-1 and Alzheimer's disease

Detection of Herpes Simplex Virus Type-1 in the Brain

Several epidemiological studies (Table 2) identified HSV1 or HSV1 DNA or HSV1 proteins or HSV1 gene sequences in the brain of Alzheimer's patients and the elderly population at high risk of developing AD.

**Table 2:** Studies demonstrating the presence of HSV1 DNA in the brain of elderly and AD patients Key: HSV1 – Herpes simplex virus type-1, DNA – Deoxyribonucleic acid, AD – Alzheimer's disease, PCR – Polymerase chain reaction

Sr. No.	Discovery	Author (Reference)	Year of Publication
1.	An autopsy study on brains of AD patients and healthy controls (elderly) found:	Harris et al. [7]	2015
		Devanand et al. [17]	2018
1a.	In the AD group, 90% of amyloid plaques contained HSV1 DNA.		
1b.	In the AD group, 72% of HSV1 DNA was plaque-associated.		
1c.	The comparison group of healthy aged brains contained fewer plaques and in the control group, only 24% of HSV1 DNA was plaque-associated (p < 0.001).		
2.	In AD patients, 90% of amyloid-β plaques were found to be co-localizing with HSV1 DNA.	Mawanda et al. [2]	2013
		Harris et al. [7]	2015
		Itzhaki et al. [8,22,32,33]	2012,2014,2017,2018
		Piacentini et al. [11]	2014
		Devanand et al. [17]	2018
		Mangold et al. [19]	2019
		Ezzat et al. [21]	2019
		Limongi et al. [25]	2016
		Lopatko et al. [26]	2019
		Wozniak et al. [27,46]	2011,2013
		La Rosa et al. [30]	2019
		Bourgade et al. [34]	2014
		Eimer et al. [37]	2018
3.	Latent HSV1 is present in a high proportion (70–100%) of sporadic AD (using PCR).	Harris et al. [7,15]	2015,2018
		Itzhaki et al. [32]	2017
4.	Presence of HSV1 DNA in human brains.	Harris et al. [7]	2015
		Itzhaki et al. [22,33]	2014,2018
5.	Detection of HSV1 DNA in a high proportion of clinically diagnosed AD brains and elderly brains.	Agostini et al. [3]	2016
		Ball et al. [20]	2012
		Limongi et al. [25]	2016
		Lopatko et al. [26]	2019
		Wozniak et al. [46,47]	2013,2013
6.	HSV1 DNA in 67 out of 70 human brains of diagnosed Alzheimer's patients.	Harris et al. [7]	2015
		Ball et al. [20]	2012
7.	In situ hybridization of postmortem brain tissue samples from 21 patients with AD and 19 controls detected HSV1 DNA in a significantly higher proportion of AD samples (81%) than controls (47.4%).	Mawanda et al. [2]	2013
8.	Detection of latent HSV1 DNA in about 60% of brains of older adults, especially in the regions critically involved in AD.	Mawanda et al. [2]	2013
		Piacentini et al. [11]	2014
		Wozniak et al. [27]	2011
9.	Detection of HSV1 DNA in several brain regions, including the hippocampus, in AD patients, and the controls.	Mawanda et al. [2]	2013
		Harris et al. [15]	2018
		Lathe et al. [24]	2019
		La Rosa et al. [30]	2019
		McManus et al. [41]	2017
10.	Demonstrated HSV1 proteins' presence in hippocampal neurons of mice infected intraperitoneally with HSV1.	Piacentini et al. (Burgos et al.) [11]	2014
11.	Detection of HSV1 DNA in the frontal and temporal cortex of AD patients.	Mawanda et al. [2]	2013
		Itzhaki et al. [8]	2012
		Harris et al. [15]	2018
		Wozniak et al. [27]	2011
		La Rosa et al. [30]	2019
12.	Detection of HSV1 thymidine kinase gene sequences in a higher proportion of brain tissue samples from AD cases (14/21) than controls (9/15) (using PCR).	Mawanda et al. [2]	2013
13.	Detection of viral DNA sequences or viral antigens and intranuclear inclusion bodies in astrocytes obtained from human brains who suffered from AD.	Martin et al. [14]	2011
14.	Features of AD pathology are transmissible by inoculation in mice and primates by HSV1.	Devanand et al. [17]	2018
15.	HSV1 DNA was identified in the trigeminal ganglion in 90% of clinical AD patients.	Devanand et al. [17]	2018
16.	In a study, 8,362 subjects aged 50 years or over during the year 2000 who were newly diagnosed with HSV1 were included.	Itzhaki et al. (Tzeng et al.) [33]	2018
16a.	The control group of 25,086 age and gender-matched subjects had no HSV infection during the year 2000.		
16b.	The frequency of dementia in the two groups was investigated during the 10 years: 2001–2010.		
16c.	The risk of developing senile dementia in the HSV group was found to be 2.56-fold greater (95% CI 2.351–2.795; P < 0.001).		

HSV1 DNA is detected in the cerebrospinal fluid, suggesting that replication occurs in the CNS [11]. HSV1 receptors are abundantly expressed in the hippocampus [24].

Role of Apolipoprotein-E Epsilon-4 (APOE-ε4)

APOE has various isoforms encoded by the APOE gene on chromosome 19 [6-7,10-11,23,30]. One of these isoforms is APOE-ε4, a well-established risk factor for AD, which when present in combination with HSV1 increases the risk for AD by 12 [2,7,10-11,14-15,17-19,22,26-27,29-30,32-33,35,41-42,46-47]. APOE plays a crucial role in the metabolism, circulation, and distribution of lipids [6-7,11,14,23]. APOE-ε4 increases the susceptibility to infiltration of the brain by HSV1 [4,7,11,14-15,19,23-24]. It enhances the attachment and entry of HSV1 into the host cells [2,7,41]. It is also responsible for increased viral load in the brain and enhances amyloid-β accumulation, thereby influencing plaque formation [7,11,15,17,41]. The risk for Alzheimer's disease increases when HSV1 is present in the brains of APOE-ε4 carriers (OR 2.71) [35]. In almost 60% of cases, HSV1 plus APOE-ε4 increases the risk for AD [8,33].

Association of Seropositivity and Cognitive Decline

HSV1 reactivation, measured by anti-HSV1 immunoglobulin M (IgM) antibodies, is associated with an increased risk of developing AD (Table 3) [3,7,13-17,20-22,25,29,42,48].

**Table 3:** Serological studies demonstrating the relationship between HSV1 and AD * Among the 43 IgM-positive subjects, only two were IgG-negative, which supports recent HSV reactivation rather than primary infection as the cause of AD in most IgM-positive subjects. Key: HSV1 – Herpes simplex virus type-1, AD – Alzheimer's disease, Ig – Immunoglobulin

Sr. No.	Study	Author (Reference)	Year of Publication
1.	The study included 3,432 elderly patients (53.9% women, mean age at inclusion 62.7 ± 14.4 years) with an average follow-up time of 11.3 years. They observed a baseline increased serum level of anti-HSV IgM antibodies associated with an increased risk of developing AD (Hazard ratio: 1.96, p = 0.012).	Harris et al. [7]	2015
		Devanand et al. [17]	2018
		LÖvheim et al. [48]	2014
2.	A population-based cohort study that followed 512 initially dementia-free older individuals for 14 years. After controlling for age, gender, educational level, and APOE-ε4 status, they found that anti-HSV1 IgM antibody seropositivity was associated with a significantly increased risk of developing AD (Hazard ratio: 2.55). *	Mawanda et al. [2]	2013
		Harris et al. [7,15]	2015,2018
		Piacentini et al. [11]	2014
		Ball et al. (Letenneur et al.) [20]	2012
3.	A prospective study performed on people over 65 years old for 12 years provided the most persuasive evidence of an association between HSV1 infection and AD progression, considering the presence of anti-HSV IgM antibodies as individuals with viral reactivation episodes.	Acuña-Hinrichsen et al. (Letenneur et al.) [31]	2019
4.	In a study, a high level of HSV1 antibodies in patients with AD was found to correlate with cortical atrophy of the gray matter using magnetic resonance.	Agostini et al. [3]	2016
		Harris et al. [7]	2015
		Acuña-Hinrichsen et al. (Mancuso et al.) [31]	2019
5.	In a study, a high level of HSV1 antibodies in patients with AD was found to be correlated with cognitive impairment evaluated through clinical tests that evaluate mental capacities (MMSE).	Harris et al. [7]	2015
		Acuña-Hinrichsen et al. (Kobayashi et al.) [31]	2019

Anti-HSV avidity index can assess HSV1 reactivation, which occurs in prodromal AD and correlates with MCI symptoms [3,7,11,17]. Measuring HSV1 antibody avidity in serum, which is a simple, non-invasive test, could help with the disease prognosis [3]. Elevated HSV1 antibody titers equated with cortical bilateral temporal and orbitofrontal gray matter volume- an indicator of AD pathology [3,4,11]. Anti-HSV IgM levels correspond inversely with lower plasma amyloid-β levels [2,11]. Lower amyloid-β in plasma indicates increased amyloid deposition in the brain, a biomarker of AD [2,11].

The hypothesis that relates HSV1 to AD's pathogenesis has gained relevance because of the detection of viral DNA and viral proteins in the brains of AD cases. Additionally, serological studies show that HSV1 reactivation is associated with an increased risk of AD. These studies prove that APOE-ε4 is a multiplying factor that increases the risk of recurrence of HSV1, invasion of CNS by HSV1 on reactivation, and developing AD in general. Thus, if HSV1 and APOE-ε4 are present together, the risk of acquiring AD increases exponentially.

Herpes simplex encephalitis and Alzheimer's disease

**Figure 4:**
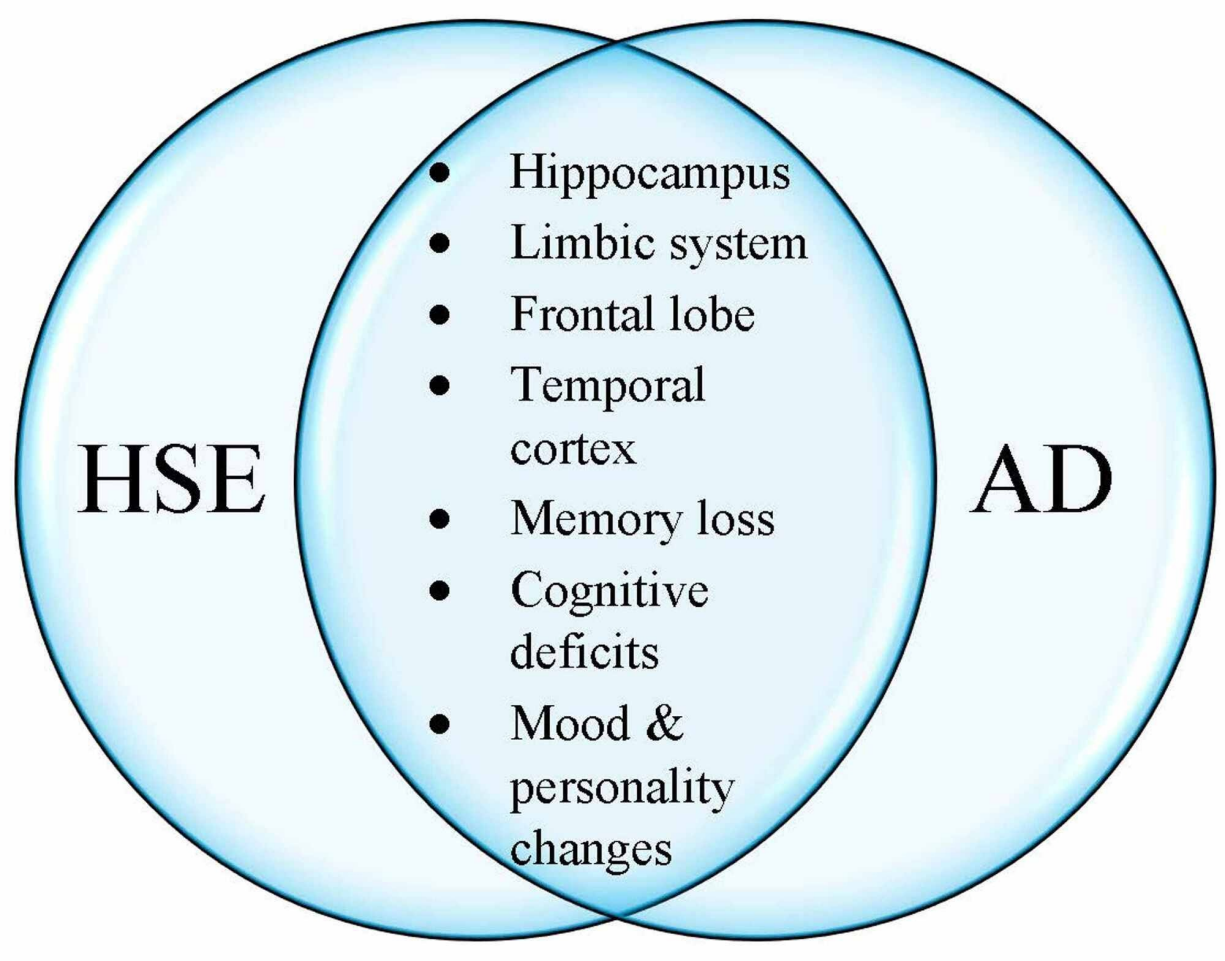
Herpes simplex encephalitis (HSE) affects the same anatomical locations in the brain, as involved in Alzheimer’s disease (AD) HSE patients are known to suffer long-term cognitive and behavioral symptoms similar to those seen in AD. HSE and AD patients have increased P-tau levels in cerebrospinal fluid (CSF). Reference: [4-5,7-8,11,14-17,19-20,27,30-31,44]

Another evidence that links HSV1 and Alzheimer's is the striking similarity between AD and HSE, a neurological condition caused by HSV1 (Figure 4).

Effect of anti-viral treatment (AVT) on Alzheimer's disease

Researchers observed that the production of P-tau depends directly on HSV1 replication or a protein depending on viral DNA replication [8,20,22,27,46-47]. In contrast, amyloid-β production is dependent on viral spread only [8,22,27,46]. The drug inhibiting viral DNA replication (ultimately curtailing its spread) decreases P-tau production and amyloid-β accumulation (Table 4) [47,49]. These drugs could impede brain degeneration and prove to be therapeutic for AD [4].

**Table 4:** Relative risks for the development of senile dementia in HSV cases and after anti-viral treatment Key: HSV1 – Herpes simplex virus type-1, SD – Senile dementia, AVT – Anti-viral treatment

Sr. No.	HSV1 infection and senile dementia (SD)	Relative risk	Author (Reference)	Year of Publication
1.	Developing SD within 10 years of HSV diagnosis vs. HSV-negative subjects	2.564	Itzhaki et al. [33]	2018
2.	Developing SD in AVT-treated HSV patients vs. untreated HSV patients	0.092	Itzhaki et al. [33]	2018

**Figure 5:**
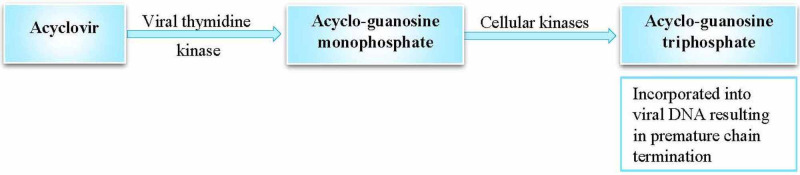
Mechanism of action of acyclovir Reference: [7,22,27]

Acyclovir, a nucleoside analog, targets infected cells and inhibits HSV1 replication and reactivation (Figure 5) [7,11,27].

**Figure 6:**
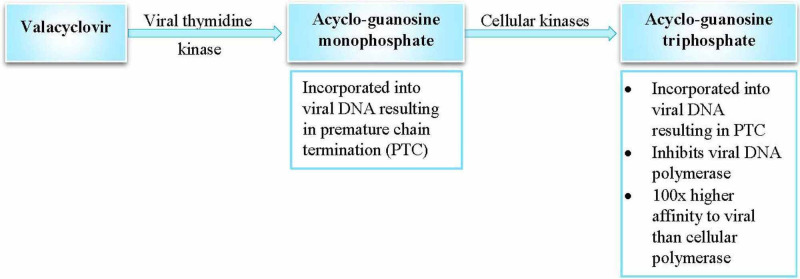
Mechanism of action of valacyclovir Reference: [17]

Valacyclovir is the bio-drug of acyclovir [7-8,17,22]. Figure 6 shows the mechanism of action of valacyclovir. Valacyclovir is rapidly hydrolyzed to acyclovir via the first-pass metabolism following oral administration [7]. The sustained activity of valacyclovir suggests that it may be symptomatic in the short-term and disease-modifying in the long-term, as per Devanand et al. [17]. They also mention a new trial of valacyclovir in the treatment of AD [17]. Penciclovir and foscarnet inhibit viral DNA replication [7].

Table 5 displays certain advantages of acyclovir and valacyclovir.

**Table 5:** Advantages of acyclovir and valacyclovir *Renal problems and crystalluria have been reported as the only side-effects by Itzhaki et al. [8]

Sr. No.	Drug	Advantages	Author (Reference)	Year of Publication
1.	Acyclovir	Crosses blood-brain barrier (BBB)	Harris et al. [7]	2015
		Affects infected cells only	Harris et al. [7]	2015
			Itzhaki et al. [8]	2012
			Piacentini et al. [11]	2014
		Safe for long term use	Harris et al. [7]	2015
2.	Valacyclovir	Crosses BBB after converting into acyclovir	Harris et al. [7]	2015
			Piacentini et al. [11]	2014
		Affects infected cells only	Harris et al. [15]	2018
			Devanand et al. [17]	2018
		Safe for long term use	Harris et al. [7,15]	2015,2018
			Devanand et al. [17]	2018
		Inexpensive	Itzhaki et al. [8]	2012
		Better oral bioavailability	Harris et al. [7]	2015
			Itzhaki et al. [8,22]	2012,2014
			Piacentini et al. [11]	2014
		Low side-effect profile*	Itzhaki et al. [8]	2012
			Harris et al. [15]	2018

By preventing HSV1 spread and its replication, anti-viral agents would provide efficacious treatment (Table 6) [27]. A study by Lathe et al. demonstrated that treatment with AVT prevented almost 90% of cases from the development of AD [24]. While HSV1 is unlikely to be the sole cause of AD, an AVT must be evaluated, primarily due to the limited effects of existing treatments and the failure of new treatments tested in patients with AD within the last two decades [17]. A study by Lin et al. about a mixed glycoprotein HSV1 vaccine has proven valuable in reducing HSV1 in the mouse brain after peripheral infection [7,15].

Intravenous immunoglobulin (IVIG) has shown anti-viral activity against HSV1 [4,7,22,46]. It neutralizes the extracellular virus [7,22,46]. Additionally, IVIG, in conjunction with lymphocytes, can destroy cells infected with HSV1 [7,22,46]. It prevents viral entry into cells and acts synergistically with acyclovir [7,22,46]. Thus, the combination of IVIG and acyclovir may be beneficial in treating AD [4,7,22,46]. Another mechanism of action of IVIG is via anti-β-amyloid antibodies, which facilitates amyloid-β clearance (Table 6) [22,46].

**Table 6:** Effect of anti-viral drugs and IVIG (intravenous Immunoglobulin) * Although neither study shows definitely that these viruses cause Alzheimer’s disease, data from a population-wide health database in Taiwan have been used to suggest not only that HSV infection increases the risk of developing the condition, but also that people treated with antiviral drugs are 10 times less likely to develop Alzheimer’s disease.

Sr. No.	Drug	Action on A-beta, P-tau, viral particles and dementia	Author (Reference)	Year of Publication
1.	Acyclovir, penciclovir, foscarnet	Decreases A-beta, P-tau, and viral particles	Devanand et al. _[17]_	2018
			Itzhaki et al. _[22]_	2014
2.	Acyclovir, valacyclovir	Decreases A-beta and P-tau	Harris et al. _[7]_	2015
3.	Anti-herpetic drugs	Decreased risk of dementia	Ezzat et al. _[21]_	2019
			Lopatko et al. _[26]_	2019
			Itzhaki et al. _[33]_	2018
			Eimer et al. _[37]_	2018
			Rizzo et al. _[40]_	2020
4.	Anti-viral drugs	Decreases A-beta, P-tau and slows or halts AD progression	Itzhaki et al. _[32,49]_	2017,2016
5.	Acyclovir	Decreases A-beta, P-tau, and viral particles	Itzhaki et al. _[8]_	2012
			Piacentini et al. _[11]_	2014
6.	Penciclovir, foscarnet	Decreases A-beta and P-tau	Harris et al. _[7]_	2015
			Wozniak et al. _[27]_	2011
7.	Anti-viral drugs	People treated with antiviral drugs are ten times less likely to develop Alzheimer’s disease*	Makin et al. _[18]_	2018
8.	Intravenous immunoglobulin (IVIG)	Decreases A-beta, P-tau, and viral particles	Agostini et al. _[4]_	2014
			Harris et al. _[7]_	2015
			Wozniak et al. _[46]_	2013

Itzhaki et al. and Wozniak et al. mention a drug, BAY57-1293, more efficient than acyclovir in inhibiting HSV1 replication and decreasing amyloid-β and P-tau formation [22,47]. It also diminishes the size of cell clusters formed during infection much more efficiently than acyclovir, suggesting that BAY57-1293 is a more effective agent for treating AD [22,47].

Sulfated fucans from five algae with anti-viral activity (Scytothamnus australis, Marginariella boryana, Papenfussiella lutea, Splachnidium rugosum, and Undaria pinnatifida) has been studied [50]. Four sulfated fucan extracts prevented the accumulation of amyloid-β and P-tau in HSV1-infected Vero cells [3,50]. The most active sulfated fucan combined with acyclovir was incredibly useful, so it may be suitable for further experimental testing to develop AD patients' treatment protocols to slow or stop disease progression [50].

The above data provide indirect evidence correlating HSV1 to AD; the similarities in the anatomical locations and the long-term symptoms seen in HSE cases and AD are too substantial to neglect. The studies demonstrating improvement in AD patients on anti-viral therapy further supports our theory that HSV1 is a potential etiological factor in AD. Acyclovir and valacyclovir have proven to be the safest option amongst anti-herpetic drugs. The above studies have shown that substances with anti-viral properties are also useful in AD. This additionally strengthens our hypothesis of viral infection being a causative factor in AD. If future researchers can provide firm evidence associating the two, newer treatment and preventive alternatives can be developed, thereby enhancing the prognosis of AD and lowering the economic burden.

Limitations

The article is a narrative review, and it, therefore, does not follow the standard Preferred Reporting Items for Systematic Reviews and Meta-Analyses (PRISMA) guidelines for systematic reviews. The possibility of bias remains both within individual studies and across studies since we could not perform a full quality assessment. We were not able to access all the articles completely, so some omissions and oversimplifications are possible.

## Conclusions

The deleterious consequences of HSV1 infection imitate the vital aspects of AD pathophysiology. Numerous studies have enlisted mechanisms used by HSV1 to prompt chief processes involved in the formation of unique signs of AD, namely, amyloid plaques and neurofibrillary tangles. Co-localization of viral DNA with amyloid plaques, similarities in the anatomical locations involved, and serological studies linking the reactivation of the virus to AD signify HSV1 as one of the causative agents of AD. There is substantial evidence proving the efficacy of anti-viral agents in the treatment and deferral of AD. Further research establishing a causative link between HSV1 and AD are needed. Interventional clinical trials for a human HSV1 vaccine and a precise anti-viral regime for preventing and treating HSV1-infected MCI and AD patients are warranted. Until we establish new therapies, frequent screening and vaccination are vital in preventing the infection-related decline of cognition.
